# Micro-Sphere PDMS for Enhancing Light Extraction in Organic Light-Emitting Devices

**DOI:** 10.3390/nano12122007

**Published:** 2022-06-10

**Authors:** Eun-Jeong Bae, Hyeong-Kyu Maeng, Ji-Soo Shin, Dong-Wook Park, Young-Wook Park, Dong-Hyun Baek

**Affiliations:** 1 Nano and Organic-Electronics Laboratory, Sun Moon University, Asan 31460, Korea; baeej2@sunmoon.ac.kr; 2 Display and Semiconductor Engineering Department, Sun Moon University, Asan 31460, Korea; tlswltn1576@naver.com; 3 Display and Nanosystem Laboratory, Department of Electrical Engineering, Korea University, Seoul 02841, Korea; 4 Physics and Nano-Science Department, Sun Moon University, Asan 31460, Korea; mmmhg@naver.com; 5 Center for Next Generation Semiconductor Technology, Department of Display and Semiconductor Engineering, Sun Moon University, Asan 31460, Korea; 6 Electrical and Computer Engineering Department, University of Seoul, Seoul 02504, Korea; dwpark31@uos.ac.kr

**Keywords:** organic light emitting-diodes (OLEDs), light extraction, breath figure (BF), sphere, out-coupling efficiency

## Abstract

We present a micro-sphere PDMS film to improve the external quantum efficiency (EQE) in OLEDs. The micro-sphere PDMS film was fabricated with the breath figure (BF) and replica molding process. The polymer template was prepared through stabilization of the water droplets at the polymer/water interface. The micro-sphere PDMS film was fabricated by pouring PDMS on the polymer template. At a 45 mg/mL concentration, the size of the spheres was approximately 12.3 µm and they had the most circular shape, so this condition yielded the best performance, with an improvement of 33% in the EQE and the widest viewing angle ranging from 0° to 50°. As a result, the sphere film’s size and distribution seem to play important roles in enhancing the EQE in OLEDs. Furthermore, the flexible sphere film based on polymeric materials could offer an effective, large-scale, mass-produced product and a simple process and approach to achieve high efficiency in flexible OLEDs.

## 1. Introduction

Organic light-emitting diodes (OLEDs) have demonstrated many advantages in flat-panel displays and lighting sources due to their high brightness, wide viewing angle, light weight and lower power consumption. They are in the spotlight as a next-generation display technology [1,2,3,4]. OLEDs have demonstrated internal quantum efficiency of nearly 100%. However, one of the main drawbacks of OLEDs is that the external quantum efficiency (EQE) is still limited because the light becomes trapped in the glass substrate, such as in substrate mode, due to the significantly different reflective indices between a transparent anode such as indium tin oxide (ITO) [5] and a glass substrate, which affect factors such as the total internal reflection (TIR) [6]; meanwhile, the organic layer and metal cathode electrode interface are attributed to the surface plasmon polariton mode. Furthermore, all the trapped light in the internal device is absorbed by the organic layers to generate a heating source; thus, the lifetime of an OLED can be degraded [7]. Indeed, the light out-coupling efficiency for OLEDs is typically limited to approximately 20%. Consequently, the low out-coupling efficiency prevents the achievement of a high EQE in OLEDs.

Many different approaches have been presented to enhance the light out-coupling efficiency in OLEDs, including microlens arrays, random micro/nanostructures, buckling patterns, the development of new emission layer materials and anti-reflection coatings. Previously reported microlens arrays with various geometries, including hemispherical [8], cylindrical [9,10] and pyramidal [11], have already been employed for the purpose to improving the light out-coupling. However, researchers have used many different technologies to produce various sizes or geometric structures with traditional photolithography, such as photoresists and transfer processes or molding. These approaches require considerably complex and costly equipment.

The breath figure (BF) process can be used to easily produce well-arranged devices that range from some hundreds to microns of diameter in hemispherical cavities [9]. Recently, several researchers have reported on different microlens arrays obtained by replica molding in the BF process [12,13,14]. These can be produced on a large scale and mass-produced, and the process is less restricted by equipment than other processes. Furthermore, polydimethylsiloxane (PDMS) has excellent formability because it has low surface energy [15]. Large-scale replica molding via the BF process can be applied to develop large-area light extraction films.

In this study, we demonstrate the enhancement of the external light extraction efficiency of OLEDs with an ordered micro-sphere PDMS film. The polymer mold is produced using the soft lithography process, breath figure (BF) process and cost-effective fabrication process. Then, the micro-sphere PDMS film is fabricated with the replication process. The dimension of the sphere is dependent on the polymer concentration. The EQE characteristics changed according to the various current densities. For example, at 22.5 mg/mL, the EQE increased by 14%, and at 45 mg/mL and 67.5 mg/mL, the EQE increased by 33%, 29%, respectively, compared to the reference condition (bare OLEDs) at the current density of 20 mA/cm^2^. The best performance was provided by 45 mg/mL, suggesting that a smaller size distribution and shape can lead to improvements in the EQE.

## 2. Materials and Methods

### 2.1. Materials

A humidifier generated moist air to control the relative humidity, and the water used in the humidifier was de-ionized water at 18 MΩ, obtained from Shinhan Science Tech (Daejeon, Korea). Polyimide film tape was purchased from 5413 3M™ Kapton^®^(supplied Dupont, Wilmington, IL, USA) and it was placed on the carrier substrate. All other reagents, namely chloroform (CHCl_3_), polystyrene (PS), polydimethylsiloxane (PDMS) prepolymer and curing agent, were analytically pure and purchased from Sigma-Aldrich (Seoul, Korea) and Dow Corning (Midland, TX, USA) (Sylgard 184).

### 2.2. Preparation of Polymer Solution and Micro-Sphere PDMS Film

The polymer solution was mixed with PS and chloroform with weight percent in a glass vial and vertex mixer for 1 h at room temperature. Then, 100 µL of polymer solution was dispensed on a glass substrate attached with polyimide tape. Then, the solvent started to evaporate in a 70% relative humidity (RH) home-made chamber, and moist air condensed onto the coated polymer solution surface simultaneously.

Then, 100 µL of polymer solution in a highly volatile solvent, namely chloroform, was dropped onto a glass substrate attached with polyimide tape under 70% RH. The fast evaporation of the solvent temporarily cooled down the solvent/air interface. This mechanism induced the condensation of water from the humid air so that the water droplets could be arranged in a hemispheric geometry. After the complete evaporation of the solvent and condensed water, the PS-based polymer mold (master mold) was formed with a micro-sphere structure. All the processes were performed at room temperature.

**Figure 1:**
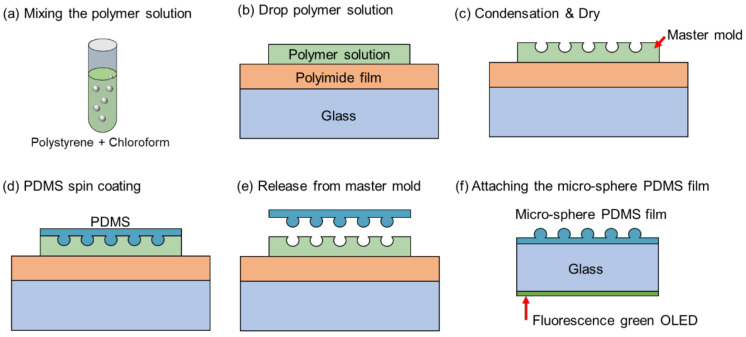
Schematic illustration of polymer mold and micro-sphere PDMS film fabrication process; (**a**) the polymer solution was produced by dissolving various forms of polystyrene (PS) in chloroform solution; (**b**) it was used as a carrier substrate by attaching polyimide tape to cleaned glass, and then 100 µL of polymer solution was dropped onto the carrier substrate at 70% RH; (**c**) after solvent application, condensed air evaporated completely, and the master mold was completed; (**d**) the uncured PDMS was poured into the polymer mold and cured at 70 °C on a hot plate for 2 h; (**e**) the micro-sphere PDMS film was released from the master mold; (**f**) the fabricated micro-sphere PDMS film was placed on the outside of the green OLED device.

The mixture of PDMS solution with 10:1 wt% was directly poured on top of the master mold and then spin coating was performed at 500 rpm to ensure the uniform thickness of the PDMS solution. Then, it was cured on a hot plate at 70 °C for 2 h. After fully curing, the micro-sphere structure on the PDMS film was replicated from the polymer mold and detached from the substrate. Finally, the spin-coated PDMS could easily infiltrate into the hemisphere structure at the master mold to give rise to a positive hemisphere structure on the PDMS film. This process is summarized in Figure 1. The image analysis software ImageJ was used to assess the size distribution in order to characterize the micro-spheres on the PDMS film. The surface morphologies and cross-sections of micro-spheres on the PDMS film were measured with scanning electron microscopy (mini-SEM, EM30, COXEM Co., Daejeon, Korea).

### 2.3. OLED Device Preparation and Characterization

The glass substrate was cleaned with an ultrasonic device for 15 min. After cleaning, the glass substrate was dried in an oven at 120 °C for 1 h. The emission area of the OLEDs was defined using a photoresist (AZ GXR 601, AZ Electronic Materials CO., Ltd., Hsinchu, Taiwan) with a size of approximately 30 mm^2^. The glass substrate was treated with UV ozone for 30 min (UVC-300, Omniscience, Gyeonggi-do, Korea) and O_2_ plasma at 60 W for 160 sec (CUTE, Femto Science Co., Hwaseong-si, Korea) to enhance the device’s emission uniformity by removing the residual contaminants. Here, 185 nm of ITO served as an anode, 60 nm of N,N′-Bis(naphthalen-1-yl)-N,N′-bis(phenyl)benzidine (NPB) served as a hole transport layer, 60 nm of Tris(8-hydroxyquinoline)aluminum (Alq_3_) served as an emission–electron transport layer, 1 nm of lithium fluoride (LiF) served as an electron injection layer, and 150 nm of aluminum (Al) served as a cathode. All the organic materials and the metal were deposited by using the thermal evaporation process at a high vacuum pressure of ~10^−7^ Torr. The deposition rates of organic materials and Al were ~1 Å/s and ~3 Å/s, respectively. Finally, the micro-sphere PDMS film was attached to the outside of the glass substrate.

To evaluate the electrical properties, the electroluminescent (EL) intensity of the micro-sphere PDMS film was measured using a spectroradiometer (CS-2000, Konica Minolta Co. Ltd., Chiyoda, Tokyo, Japan) in a dark box, and a source meter (2410, Keithley, Tektronix, Beaverton, OR, USA) was applied at a driving voltage. The EL characteristics, including the viewing angle, were directly measured with a spectroradiometer. The external quantum efficiency (EQE) was calculated assuming that the emitted light of OLEDs was in the form of Lambertian emission, and then the EQE was calculated for the measured area with the various viewing angles recorded (10–70°).

## 3. Results and Discussion

To form a rugged structure to enhance the light extraction of OLEDs, we fabricated a polymer mold, which was subjected to condensed moist air and solvent evaporation on the surface of the coated polymer solution using the breath figure process (BF). After this, the uncured PDMS was poured into the polymer mold, and the micro-sphere film was fabricated with the replication process.

**Figure 2:**
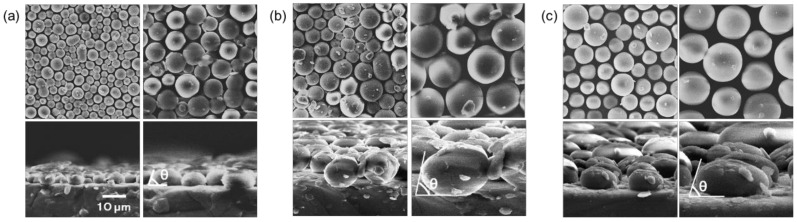
The scanning electron microscopy (SEM) images of the micro-sphere PDMS film: (**a**) 22.5 mg/mL concentration; (**b**) 45 mg/mL concentration; (**c**) 67.5 mg/mL concentration; (**top**) top-view, (**bottom**) cross-sectional view and magnified sphere shape for determination; scale bar shows 10 µm.

The morphology and cross-section of the micro-sphere PDMS film is shown in Figure 2. The diameter of spheres differed depending on the PS concentration in the polymer solution ((a) 22.5 mg/mL, (b) 45 mg/mL, (c) 67.5 mg/mL). The spheres were obtained with diameters ranging from approximately 1 to 25 µm, but the spheres showed high circularity. Here, an analysis of the sphere size was performed using the commercially available ImageJ. Figure 2a shows the uniformly covered and highly ordered structure, and (b) shows a less ordered array than (a) due to its wider range of dimension. On the other hand, (c) is the largest structure, with a poorly ordered arrangement.

**Figure 3:**
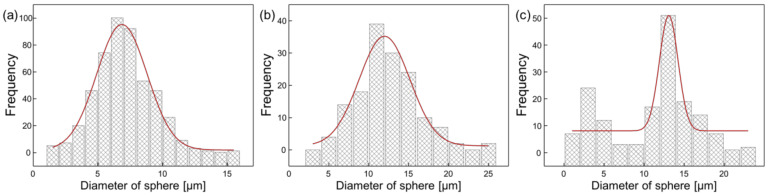
The size distribution of micro-spheres on PDMS film depending on the polymer concentration: (**a**) 22.5 mg/mL concentration (*n* = 5); (**b**) 45 mg/mL concentration (*n* = 5); (**c**) 67.5 mg/mL concentration (*n* = 5).

Xi et al. and Hu et al. found that the formation of condensed water and the wettability of the carrier substrate are important for the formation of a micro-sphere structure [16,17], so we used a polyimide film to maintain the wettability of the surface. In particular, polystyrene (PS), an amphiphilic polymer, is known to have an important characteristic that helps to form a well-ordered structure through the stabilization of the water droplets at the polymer/water interface [18]. It is noteworthy that the average sphere sizes of the obtained PDMS film were 7.00 ± 2.10 µm, 12.3 ± 3.71 µm and 11.1 ± 5.34 µm when the PS concentrations in the polymer solution increased, as illustrated in Figure 3. Analysis of the size distribution revealed the effect of the PS concentration on the formation of the micro-sphere PDMS film. Depending on the polymer used and the conditions employed, it was possible to control both the size and the degree of order.

**Figure 4:**
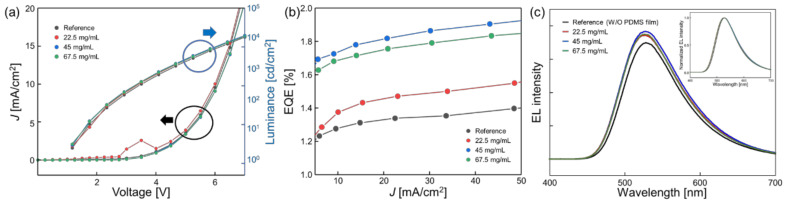
The electroluminescent (EL) characteristics of OLEDs depending on the micro-sphere PDMS film; (**a**) current density–voltage–luminance curve (The black circle is the current density of the left Y-axis, The blue circle is the luminance of the right Y-axis); (**b**) current density–EQE curve (red: 22.5 mg/mL, blue: 45 mg/mL, green: 67.5 mg/mL PS concentration in polymer solution); (**c**) EL spectra of the OLED device with or without each micro-sphere PDMS film with 20 mA/cm^2^ (the inset graph shows the normalized EL spectra).

Figure 4 shows the EL characteristics of the fabricated OLEDs with and without the micro-sphere PDMS film. The voltage–current density curve shows that the electrical property is almost equivalent with and without micro-sphere PDMS films because the OLED is stably operated, as shown in Figure 4a. Figure 4b shows the EQE characteristics according to the various current densities. At 22.5 mg/mL, the EQE increases by 14%. Moreover, at 45 mg/mL and 67.5 mg/mL, the EQE increases by 33% and 29%, respectively, compared to the reference condition (bare OLEDs) at the current density of 20 mA/cm^2^. The 45 mg/mL and 67.5 mg/mL conditions result in a similar size of sphere, but the size range is narrower than at the 67.5 mg/mL concentration. Figure 4c shows the EL spectra of the OLEDs. A value of 20 mA/cm^2^ was applied to the state of the micro-sphere PDMS film. An identical peak was observed at a wavelength of 525 as the emission peak. A spectral difference and peak wavelength difference did not appear. As a result, the sphere size and distribution of size seem to play important roles. This result indicates that the sphere size, shape and distribution significantly affect the EL characteristics of applied OLEDs. The best performance is provided by 45 mg/mL, suggesting that a smaller size distribution and shape can affect the improvement in the EQE, as shown in Table 1.

**Figure 5:**
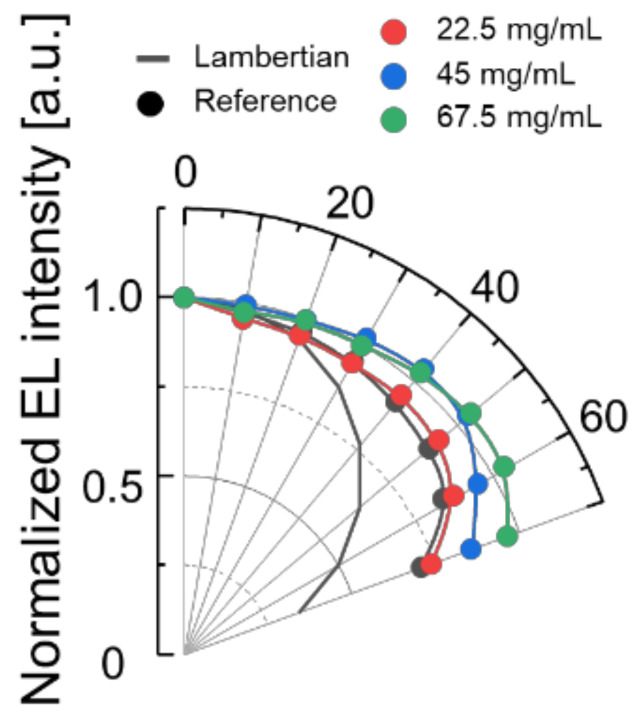
The normalized angular intensity distribution (black line: theoretical Lambert distribution).

In order to determine the origin of the light extraction enhancement, the EL intensity according to viewing angles ranging from 0° to 70° was measured with the fabricated OLEDs with or without the micro-sphere PDMS film, and the normalized angular intensity results are shown in Figure 5. Since the luminance in the normal direction differs according to the film conditions, it is normalized based on luminance in the normal direction. Regarding the viewing angle characteristics, overall conditions show a wider profile than the Lambertian light source. The 45 mg/mL and 67.5 mg/mL concentrations show the widest profile.

## 4. Conclusions

We fabricated a flexible micro-sphere PDMS film with a simple and cost-effective process. The fabricated micro-sphere PDMS film can be easily applied to the external light extraction layer in OLEDs. The experimental results show the influence of the PS concentration on the size distribution and sphere shape, which could be controlled by the PS concentration in the polymer solution. We found that the main parameters significantly improved the light extraction with respect to the reference device (bare OLEDs), as shown in Table 1. The best performance condition was achieved with a diameter of 12.3 µm and enhancement of 33% in EQE in the proposed process. Thus, the development of micro-sphere PDMS films is highly relevant for the achievement of high-efficiency flexible OLEDs. Furthermore, the micro-sphere PDMS films are suitable for application in flexible electronics.

We believe that the present work demonstrates an effective methodology to enhance the out-coupling efficiency of OLEDs, and this approach can be also applied to large-scale and mass products. This array film, suitable for human-friendly wearable electronics, can contribute to the development of efficient wearable light sources. In the future, the micro-sphere film based on a polymeric material could provide the opportunity to achieve high efficiency in flexible OLEDs.

## Figures and Tables

**Table 1:** Summary of main parameters describing the three micro-sphere PDMS films.

Concentration of Polymer Solution (mg/mL)	Mean Diameter (µm)	SD	Enhancement (%)	ϴ (°)
22.5	7.00	2.10	14	60
45	12.3	3.71	33	71
67.5	11.1	5.34	29	67

## Data Availability

Data presented in this study is available in this article.
